# Supplementary material for: Effect of 3D Spheroid Culturing on NF-κB Signaling Pathway and Neurogenic Potential in Human Amniotic Fluid Stem Cells
Source: Int J Mol Sci. 2023 Feb 10;24(4):3584. doi: 10.3390/ijms24043584 (PMC9963588; doi:10.3390/ijms24043584)
Supplement: Supplementary file 1 [file ijms-24-03584-s001.zip › Figure S1.pdf]

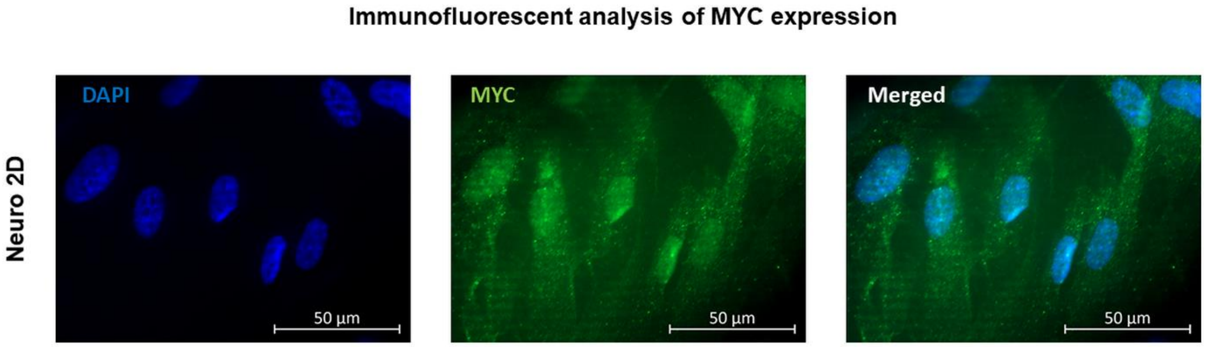

Figure S1. MYC expression profile in hAFSCs induced to neural differentiation, when cells were grown in 2D culture. Representative images of neuro-induced 2D (Neuro 2D), showing positive cells for MYC (green). Nuclei were counterstained with DAPI (blue); scale bar = 50 μm.
