# Supplementary material for: Effect of 3D Spheroid Culturing on NF-κB Signaling Pathway and Neurogenic Potential in Human Amniotic Fluid Stem Cells
Source: Int J Mol Sci. 2023 Feb 10;24(4):3584. doi: 10.3390/ijms24043584 (PMC9963588; doi:10.3390/ijms24043584)
Supplement: Supplementary file 1 [file ijms-24-03584-s001.zip › Table S1.pdf]

Table S1. The 25 most relevant pathways from Reactome database sorted by p-value.

	Upregulated in 3D secretome		Downregulated in 3D secretome	
**No.**	**Pathway name**	**p-value**	**Pathway name**	**p-value**
**1**	Retinoid metabolism and transport	2.73E-4	Extracellular matrix organization	5.29e-13
**2**	Metabolism of fat-soluble vitamins	3.52E-4	Degradation of the extracellular matrix	3.66e-09
**3**	Plasma lipoprotein assembly	1.37E-3	Syndecan interactions	1.31e-08
**4**	Retinoid metabolism disease events	2.84E-3	Regulation of Insulin-like Growth Factor (IGF) transport and uptake by Insulin-like Growth Factor Binding Proteins (IGFBPs)	4.25e-08
**5**	Visual phototransduction	2.88E-3	ECM proteoglycans	6.19e-08
**6**	Post-translational protein phosphorylation	3.48E-3	Post-translational protein phosphorylation	4.54e-07
**7**	Regulation of Insulin-like Growth Factor (IGF) transport and uptake by Insulin-like Growth Factor Binding Proteins (IGFBPs)	5.24E-3	Non-integrin membrane-ECM interactions	6.11e-07
**8**	Formation of Fibrin Clot (Clotting Cascade)	5.57E-3	Assembly of collagen fibrils and other multimeric structures	7.19e-07
**9**	Defective SLC40A1 causes hemochromatosis 4 (HFE4) (macrophages)	5.67E-3	Collagen degradation	9.09e-07
**10**	Defective CP causes aceruloplasminemia (ACERULOP)	5.67E-3	Platelet degranulation	1.28e-06
**11**	Regulation of Complement cascade	6.63E-3	MET activates PTK2 signaling	1.52e-06
**12**	Complement cascade	8.21E-3	Response to elevated platelet cytosolic Ca2+	1.59e-06
**13**	Alternative complement activation	1.41E-2	Hemostasis	2.17e-06
**14**	Loss of proteins required for interphase microtubule organization from the centrosome	1.65E-2	Activation of Matrix Metalloproteinases	2.21e-06
**15**	Loss of Nlp from mitotic centrosomes	1.65E-2	Collagen formation	4.75e-06
**16**	PTK6 promotes HIF1A stabilization	1.69E-2	MET promotes cell motility	5.18e-06
**17**	Metabolism of vitamins and cofactors	1.71E-2	Platelet activation, signaling and aggregation	6.61e-06
**18**	AURKA Activation by TPX2	1.79E-2	Signaling by MET	6.97e-05
**19**	Plasma lipoprotein assembly, remodeling, and clearance	1.79E-2	Integrin cell surface interactions	8.80e-05
**20**	Cilium Assembly	1.95E-2	Interleukin-4 and Interleukin-13 signaling	2.43e-04
**21**	Activation of C3 and C5	1.97E-2	Collagen chain trimerization	2.49e-04
**22**	Centrosome maturation	2.23E-2	Signaling by Receptor Tyrosine Kinases	5.57e-04
**23**	Recruitment of mitotic centrosome proteins and complexes	2.23E-2	Collagen biosynthesis and modifying enzymes	8.43e-04
**24**	Transport of fatty acids	2.25E-2	Crosslinking of collagen fibrils	0.001
**25**	HDL assembly	2.25E-2	Cell-extracellular matrix interactions	0.001
