# Supplementary material for: Effect of 3D Spheroid Culturing on NF-κB Signaling Pathway and Neurogenic Potential in Human Amniotic Fluid Stem Cells
Source: Int J Mol Sci. 2023 Feb 10;24(4):3584. doi: 10.3390/ijms24043584 (PMC9963588; doi:10.3390/ijms24043584)
Supplement: Supplementary file 1 [file ijms-24-03584-s001.zip › Table S2.pdf]

Table S2. Antibodies used for flow cytometry analysis.

Antibody	Fluorophore	Host/Isotype	Manufacturer
anti-CD31	Alexa Fluor® 488	Mouse/IgG1, κ	Biolegend, San Diego, California, USA
anti-CD34	FITC	Mouse/IgG2a, κ	
anti-CD44	APC	Mouse/IgG1, κ	
anti-CD56	APC	Mouse/IgG1, κ	
anti-CD117	APC	Mouse/IgG1, κ	
anti-CD146	APC	Mouse/IgG1, κ	
anti-CD166	PE	Mouse/IgG1, κ	
anti-SSEA4	APC	Mouse/IgG3, κ	
anti-HLA-ABC	Alexa Fluor® 488	Mouse/IgG2a, κ	
anti-HLA-DR	Alexa Fluor® 488	Mouse/IgG2a, κ	
anti-CD73	FITC	Mouse/IgG1, κ	Exbio, Vestec, Czech Republic
anti-CD90	APC	Mouse/IgG1, κ	
anti-CD105	APC	Mouse/IgG1, κ	
anti-LIN28a	-	Mouse/IgG2b	Novus Biologicals, Abingdon, UK
anti-MSI1	-	Rabbit/IgG	
anti-Nestin	-	Mouse/IgG1, κ	
Secondary Anti-mouse	Alexa Fluor® 488	Goat / IgG	Invitrogen, Thermo Fisher Scientific, Waltham, MA, USA
Secondary Anti-rabbit	Alexa Fluor® 488	Goat / IgG	
