# Supplementary material for: Effect of 3D Spheroid Culturing on NF-κB Signaling Pathway and Neurogenic Potential in Human Amniotic Fluid Stem Cells
Source: Int J Mol Sci. 2023 Feb 10;24(4):3584. doi: 10.3390/ijms24043584 (PMC9963588; doi:10.3390/ijms24043584)
Supplement: Supplementary file 1 [file ijms-24-03584-s001.zip › Table S3.pdf]

Table S3. List of primers sequences for gene expression analysis.

Name	Forward primer sequence (5'-3')	Reverse primer sequence (5'-3')
GAPDH	AGTCCCTGCCACACTCAG	TACTTTATTGATGGTACATGACAAGG
RPL13A	GTTGATGCCTTCACAGCGTA	AGATGGCGGAGGTGCAG
SOX2	TGGACAGTTACGCGCACAT	CGAGTAGGACATGCTGTAGGT
OCT4	CGAGAAGGATGTGGTCCGAG	CAGAGGAAAGGACACTGGTC
NANOG	AGATGCCTCACACGGAGACT	GTTTGCCTTTGGGACTGGTG
LIN28A	TTGTCTTCTACCCTGCCCTCT	GAACAAGGGATGGAGGGTTTT
MSI1	TGAGCAGTTTGGGAAGGTG	TCACACACTTTCTCCACGATG
MSI2	ATCCCACTACGAAACGCTCC	GGGGTCAATCGTCTTGGAATC
NOTCH1	GGTGAGACCTGCCTGAATG	GTTGGGGTCCTGGCATC
MYC	AATGAAAAGGCCCCCAAGGTAGTTATCC	GTCGTTTCCGCAACAAGTCCTCTTC
TP53	TAACAGTTCCTGCATGGGCGGC	AGGACAGGCACAAACACGCACC
BAK1	TCATCGGGGACGACATCAAC	CAAACAGGCTGGTGGCAATC
BAX	TGCCTCAGGATGCGTCCACCAA	CCCCAGTTGAAGTTGCCGTCAG
MCL1	GTGCCTTTGTGGCTAAACACT	AGTCCCGTTTTGTCCTTACGA
HIF1A	CCAACAGTAACCAACCTCAG	TCCTGTGGTGACTTGTCCTT
MAP2	CCAATGGATTCCCATACAGG	TCCTTGCAGACACCTCCTCT
NCAM1	TGTCCGATTCATAGTCCTGTCC	CTCACAGCGATAAGTGCCCTC
NSE	CCCACTGATCCTTCCCGATACAT	CCGATCTGGTTGACCTTGAGCA
NES	CTGCTACCCTTGAGACACCTG	GGGCTCTGATCTCTGCATCTAC
TUBB3	CTCAGGGGCCTTTGGACATC	CAGGCAGTCGCAGTTTTCAC
NTRK1	CCCCATCCCTGACACTAACA	GCACAAGGAGCAGCGTAGAA
NTRK2	ACCCGAAACAAACTGACGAGT	AGCATGTAAATGGATTGCCCA
GFAP	GGCAAAAGCACCAAAGACGG	GGCGGCGTTCCATTTACAAT
NGF	TGTGGGTTGGGGATAAGACCA	GCTGTCAACGGGATTTGGGT
CNTF	GAAGATTCGTTCAGACCTGACTG	AAGGTTCTCTTGGAGTCGCTC
ALDH1L1	GCCTGGCTTCTGGTGTCTTC	GCCACGTCGGTCTTGTTGTA
GDNF	ACCTGGAGTTAATGTCCAACC	GGCATATTTGAGTCACTGCT
NEUROD1	ATGACCAAATCGTACAGCGAG	GTTCATGGCTTCGAGGTCGT
VEGFA	AGGGCAGAATCATCACGAAGT	AGGGTCTCGATTGGATGGCA
VIM	TCTCTGAGGCTGCCAACCG	CGAAGGTGACGAGCCATTTCC
NFKB1	GAAGCACGAATGACAGAGGC	GCTTGGCGGATTAGCTCTTTT
NFKB2	ATGGAGAGTTGCTACAACCCA	CTGTTCCACGATCACCAGGTA
RELA	ATGTGGAGATCATTGAGCAGC	CCTGGTCCTGTGTAGCCATT
REL	AAAGACTGCAGAGACGGCTA	CTCACCACATTGAGGTCACA
TNFR1	TCACCGCTTCAGAAAACCACC	GGTCCACTGTGCAAGAAGAGA
TNFR2	TTCATCCACGGATATTTGCAGG	GCTGGGGTAAGTGTACTGCC
